# Unraveling the Immune Microenvironment in Classic Hodgkin Lymphoma: Prognostic and Therapeutic Implications

**DOI:** 10.3390/biology12060862

**Published:** 2023-06-15

**Authors:** Vasileios Georgoulis, Alexandra Papoudou-Bai, Alexandros Makis, Panagiotis Kanavaros, Eleftheria Hatzimichael

**Affiliations:** 1 Department of Hematology, School of Health Sciences, Faculty of Medicine, University of Ioannina, 45 500 Ioannina, Greece; vasileios.georgoulis@gmail.com; 2 Department of Pathology, School of Health Sciences, Faculty of Medicine, University of Ioannina, 45 500 Ioannina, Greece; apapoudoubai@gmail.com; 3 Department of Child Health, School of Health Sciences, Faculty of Medicine, University of Ioannina, 45 500 Ioannina, Greece; amakis@uoi.gr; 4 Department of Anatomy-Histology-Embryology, School of Health Sciences, Faculty of Medicine, University of Ioannina, 45 000 Ioannina, Greece; pkanavar@uoi.gr

**Keywords:** Hodgkin lymphoma, tumor microenvironment, tumor associated macrophages, CD169^+^ macrophages, immune evasion, immunosuppression

## Abstract

**Simple Summary:**

Hodgkin lymphoma accounts for 10% of new lymphoma diagnoses and has generally high cure rates, although there is a need for new treatments in the relapsed setting. On the microscopic scale, neoplastic cells are believed to be in a constant cross-talk with their surrounding immune cells and shape a microenvironment that suppresses host’s anti-tumor immunity. In this review, we summarize findings regarding the role of each cell in the tumor microenvironment of classic Hodgkin lymphoma, with a focus on macrophages, and we describe ways in which the tumor cells manage to escape from the patient’s immune surveillance. Within this microenvironment, novel therapeutic targets have emerged, allowing for a personalized approach for patients with Hodgkin lymphoma.

**Abstract:**

Classic Hodgkin lymphoma (cHL) is a lymphoid neoplasm composed of rare neoplastic Hodgkin and Reed–Sternberg (HRS) cells surrounded by a reactive tumor microenvironment (TME) with suppressive properties against anti-tumor immunity. TME is mainly composed of T cells (CD4 helper, CD8 cytotoxic and regulatory) and tumor-associated macrophages (TAMs), but the impact of these cells on the natural course of the disease is not absolutely understood. TME contributes to the immune evasion of neoplastic HRS cells through the production of various cytokines and/or the aberrant expression of immune checkpoint molecules in ways that have not been fully understood yet. Herein, we present a comprehensive review of findings regarding the cellular components and the molecular features of the immune TME in cHL, its correlation with treatment response and prognosis, as well as the potential targeting of the TME with novel therapies. Among all cells, macrophages appear to be a most appealing target for immunomodulatory therapies, based on their functional plasticity and antitumor potency.

## 1. Introduction

Hodgkin lymphoma (HL) is a B cell lymphoid neoplasm accounting for approximately 10% of all new lymphoma diagnoses in the Western world. The incidence of the disease is about 2–3/100,000 with bimodal peaks of diagnoses in the third and eighth decades of life. The mortality rate of HL is 0.4/100,000 per year, depicting the high cure rates achieved with classic chemotherapy, radiotherapy and targeted immunotherapy, although the prognosis is inferior for patients at relapse [1,2].

Histologically, 95% of HL cases are termed as classic HL (cHL) with the rest of the diagnoses accounting for nodular lymphocyte-predominant HL (NLPHL), while cHL is further subdivided into four subtypes: nodular sclerosis, mixed cellularity, lymphocyte-rich and lymphocyte depletion, each with different pathologic and clinical features.

Regardless of the histologic subtypes, the pathologic hallmark of cHL is the neoplastic Hodgkin and Reed–Sternberg cells (HRS). The latter ones are bi- or multi-nucleated and arise from the mono-nucleated Hodgkin cells through incomplete cytokinesis and the re-fusion of daughter cells [3]. Given their clonal Ig rearrangements and Ig genes’ somatic hypermutations, HRS cells are believed to stem from pre-apoptotic germinal center B cells, although they have suppressed part of their B cell gene expression program. Using immunohistochemistry, HRS cells are typically positive for CD30 and variably positive for CD15 [4,5,6].

Multiple genetic mutations have been identified in HRS cells, and most of them are those that affect molecular signaling pathways regulating cell survival and proliferation, primarily the NF-κΒ, JAK/STAT, PI3K/AKT and NOTCH1 pathways [7]. Another commonly detected genetic alteration in HRS cells is the amplification of the genes programmed death (*PD)-L1* and *PD-L2*, leading to the overexpression of the encoded immune checkpoint proteins, which regulate immune evasion [8].

Although HRS cells are considered a histologic hallmark of cHL tumors, these cells are rare, representing only 1% of the cellular composition of the tumor, and are embedded within an abundant reactive cellular infiltrate. This non-neoplastic immune population is composed of T- and B-lymphocytes, neutrophils, eosinophils, macrophages, plasma cells, NK cells, dendritic cells and mast cells, which are recruited and educated by the HRS cells. Specifically, HRS cells produce several cytokines such as CC motif chemokine ligand (CCL) 5, CCL17, CCL20, CCL22, Chemokine C-X-C motif ligand (CXCL)9 and CXCL10 along with interleukin (IL)-5, IL-8 and IL-9 that promote the recruitment of the immune cells [4,9]. These reactive cells, in cooperation with stromal cells, fibroblasts and endothelial cells, shape a unique tumor microenvironment (TME) which is in close crosstalk with the neoplastic population. TME is believed to support the survival and promote the proliferation of HRS cells. Moreover, TME has been shown to have suppressive properties against anti-tumor immunity, thus allowing the immune evasion of the neoplasm through the production of various cytokines and the aberrant expression of immune checkpoint molecules [10]. In this way, the TME might be involved in treatment resistance, and, hence, it could possibly provide targets for novel therapeutic strategies.

In this review, we summarize important research findings regarding the cellular and molecular composition of the TME in cHL, with an emphasis on macrophage populations. We also shed light on a recently studied subtype of macrophages, CD169^+^, and focus on how the TME and HRS cells achieve immune evasion and how the communication between neoplastic and immune cells is correlated with disease outcomes and treatment response.

## 2. Overview of Cellular Components of TME in cHL

### 2.1. T-Cells

T cells in the TME of HL have been the object of intense investigation, among all other immune cells, due to their abundance and functional plasticity.

#### 2.1.1. CD4^+^ T-Cells

**Figure 1:**
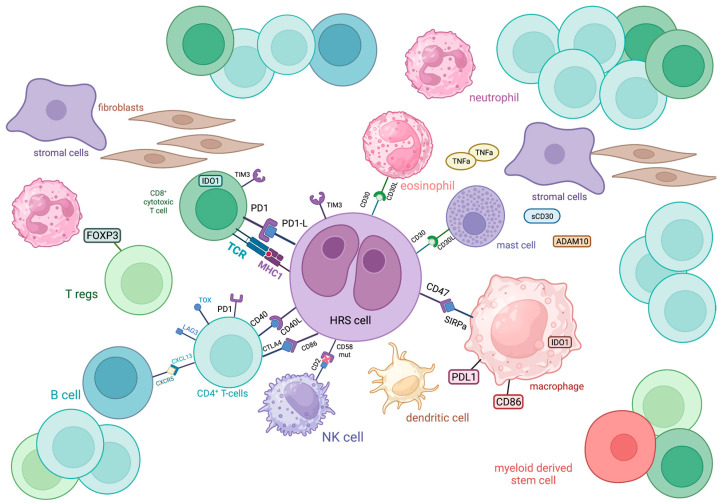
Schematic representation of the immune cellular interlay within the tumor microenvironment of classic Hodgkin lymphoma.

The most abundant cellular population in the TME of HL comprises CD4^+^ T cells, with a T-helper (T_h_) phenotype, which tend to gather around HRS cells in formations called rosettes (Figure 1). These cells express T cell exhaustion markers including PD-1, TOX and TOX2 [11]. A high percentage (>75%) of tumor-infiltrating CD4^+^ T cells was associated with decreased freedom from treatment failure (FFTF) [12]. Initially, it was believed that the TME of cHL is dominated by T_h_2 CD4^+^ T cells, with their increased number predicting improved disease-free survival (DFS) and event-free survival (EFS) [13]. T_h_2 cells are generally involved in type 2 immune response, mediated by IL-4, IL-5, IL-9 and IL-13, which participate in anti-helminthic immunity and tissue regeneration [14]. Nevertheless, more recent research revealed that T_h_ cells in the TME of HL primarily polarized towards the T_h_1 phenotype [15,16]. T_h_1 immunity is based on IL-2, interferon (IFN)-γ and tumor necrosis factor (TNF)-β and regulates cell-mediated immune reactions that also protect against tumor cells [17,18].

FOXP3^+^ regulatory T cells (T_regs_) are also present in the TME of cHL. These cells restrict T_h_-mediated immune responses through the secretion of immunosuppressive cytokines. In this way, they are involved in sustaining self-tolerance but also inhibit antitumor immunity [19]. In cHL, low numbers of FOXP3^+^ T_regs_ in TME, in combination with high numbers of cytolytic T cells, were correlated with shortened survival [20]. Additionally, the T_regs_/T_h_17 ratio in cHL patients was found to be positively associated with survival, thus implying that higher T_h_17 infiltration might characterize a more aggressive disease course [21,22]. However, this reflects peripheral blood values and not the actual TME, present in tissues affected by cHL, where more extensive research is warranted to establish the putative prognostic significance of T_regs_ in the TME of cHL.

ADAM10: a disintegrin and metalloproteinase domain-containing protein 10; CTLA4: cytotoxic T-lymphocyte-associated protein 4; CXCL13: chemokine (C-X-C motif) ligand 13; CXCR5: C-X-C chemokine receptor type 5; FOXP3: forkhead box P3, IDO1: indoleamine-pyrrole 2,3-dioxygenase 1; LAG3: lymphocyte-activation gene 3; MHC: major histocompatibility complex; PD1: programmed cell death protein 1; PDL1: programmed death-ligand 1; SIRPa: signal regulatory protein α; TCR: T cell receptor; TIM3: T cell immunoglobulin and mucin domain-containing protein 3; TNFa: tumor necrosis factor α; TOX: thymocyte selection-associated high mobility group box

Importantly, results on the prognostic significance of T_regs_ should be interpreted with caution due to limitations in identifying this specific cellular population. Although FOXP3 is considered the best marker for T_regs_ [23,24], it has been reported that human non-regulatory CD4^+^ or CD8^+^ T cells are capable of FOXP3 expression [25,26] and that most human FOXP3^−^ CD25^−^ T cells may transiently obtain the phenotype of T_reg_ upon activation, including the co-expression of FOXP3 and CD25 and the inhibition of the proliferation of autologous CD4^+^CD25^−^ T cells [27]. Additionally, the coculture of cHL cell lines (KM-H2 and HDLM-2) with peripheral blood mononuclear cells (PBMC) promoted the enrichment of Th17 lymphocytes and FOXP3^+^/IL-17^+^ cells, whereas the population of T_regs_ was slightly diminished [28], indicating that FOXP3 alone might not be adequate for the detection of T_regs_.

Single-cell expression profiling in cHL has revealed a novel subset of T cells with prominent expression of the lymphocyte activation gene-3 (LAG-3), and functional analyses provided evidence that this LAG3^+^ T cell population mediated immunosuppression [29]. In addition, multiplexed spatial analysis of immune cells in the TME disclosed increased numbers of LAG3^+^ T cells in the close vicinity of MHC class II-deficient neoplastic HRS cells [29]. The authors suggested that this immunosuppressive subset of LAG3^+^ T cells might contribute to the immune-escape phenotype [29]. In another study, single-cell RNA sequencing analysis identified a subset of CD4^+^ helper T cells in lymphocyte-rich cHL, which was characterized by high expression of PD-1 and CXCL13 [30]. These PD-1^+^CXCL13^+^ T cells were significantly enriched in lymphocyte-rich cHL compared to other subtypes of cHL and frequently formed rosettes around neoplastic HRS cells [30]. Multicolor immunofluorescence analysis demonstrated that the PD-1^+^CXCL13^+^ T cells were in close proximity to CXCR5^+^ normal B cells in lymphocyte-rich cHL [30]. In addition, high levels of PD-1^+^CXCL13^+^ T cells in the TME showed a statistically significant association with shorter progression-free survival and shorter overall survival in lymphocyte-rich cHL [30]. The authors suggested that the CXCL13/CXCR5 axis may have pathogenetic importance and that the PD-1^+^CXCL13^+^ T cells may be a potential treatment target in lymphocyte-rich cHL [30].

T cells in cHL express a variety of immune checkpoint regulators, including CTLA-4, PD-1 and LAG-3, shaping a unique immunosuppressive TME that enables HRS cells to escape antitumor immunity, as discussed later (Figure 1).

#### 2.1.2. CD8^+^ T-Cells

CD8^+^ T cells are generally known as cytolytic T cells (CTLs) due to their capacity to directly kill infected or neoplastic cells after recognizing antigens bound to MHC (major histocompatibility complex)-I molecules on their surface and are, therefore, considered as important mediators of antitumor immunity, along with other major cytolytic cells, NK cells. In the case of cHL, the CD8^+^ T cell subpopulation is less abundant than the CD4^+^ one, with contradictory results regarding its prognostic value. Alonso-Álvarez et al. found that high numbers of CD8^+^ T cells predict better outcomes in patients treated with ABVD as first-line therapy [12]. In contrast, the presence of activated CTLs (positive for TIA-1 and granzyme B) in the TME of cHL has been correlated with decreased survival in the relapsed/refractory (R/R) setting [20,31]. Regardless of their prognostic role, CD8^+^ T cells seem to have an important role in shaping the TME of cHL, since they express immune checkpoint molecules such as PD-1, indoleamine 2,3-Dioxygenase 1 (IDO)-1 and TIM-3 more frequently than CD4^+^ T cells [32] (Figure 1). However, these cells seem to have diverged from their cytotoxic role against neoplastic cells. A subset of CD8^+^ T cells was identified in cHL TME that shares phenotypic and functional characteristics with T-follicular helper cells. Specifically, they co-express CXCR5 and ICOS, Bcl-6, PD-1 and CD200 and show deficient cytotoxicity and low IFN-γ secretion [33]. The function of CD8^+^ T cells might be negatively influenced by Galectin-1 produced by HRS cells [34].

### 2.2. B-Cells

The presence and prognostic value of non-neoplastic B cells of TME in cHL has been studied by independent research groups, based on their negative impact on several solid malignancies. Interestingly, high proportions of CD20^+^ background cells in TME were correlated with increased overall survival (OS), while low B cell counts were associated with shortened progression-free survival (PFS) and OS among patients treated with BEACOPP-based regimens; thus, B cells show both a prognostic and predictive value [35,36]. A possible explanation for B cells’ favorable effect might be the competition with neoplastic cells for survival and growth signals, although more research is needed to clarify whether all B cells or specific sub-populations have a favorable predictive impact since the presence of the PAX5^+^/CD38^+^ sub-population was shown to correlate with adverse outcomes [37,38].

### 2.3. Plasma Cells

Data on the role of plasma cells in the TME of cHL has been scarce so far. Tumor infiltration by CD138^+^ plasma cells is associated with advanced disease stage, eosinophil infiltration and the presence of B symptoms and a tendency towards inferior OS and EFS [39]. Additionally, elevated polyclonal serum-free light chains in patients with cHL showed a correlation with decreased survival and it has been assumed that these light chains are produced by plasma cells of the TME since HRS cells are considered incapable of secreting immunoglobulins [40].

### 2.4. NK-Cells

Although NK cells are innate lymphoid cells with known anti-tumor cytotoxic activity, in the case of HL TME, these cells seem to be numerically and functionally diminished. The inhibition of NK cytotoxic activity is primarily mediated by ligands found in TME which bind to NK-inactivating receptors [41]. Furthermore, in patients with HL, the ratio of CD56^dim^DNAM-1^pos^ NK cells over CD56^dim^DNAM-1^neg^ NK cells is reduced, indicating a shift of the NK phenotype towards the less cytotoxic DNAM-1^neg^ population. Even CD56^dim^DNAM-1^pos^ NK cells were found to show impaired cytotoxic activity in HL patients compared to healthy individuals [42]. One possible explanation for the limited NK population in HL TME might be the induction of apoptosis triggered by the binding of the Fas-L of HRS cells to the Fas receptor of NK cells [22]. Again, these results were obtained from peripheral blood analysis, and their contribution to the understanding of the actual TME of cHL should be analyzed by comparing data from similar biological materials.

### 2.5. Myeloid Cells

Myeloid-derived suppressor cells (MDSCs) are a heterogeneous population of immature myeloid cells, expressing CD11b and CD33, which exert immunosuppressive roles when infiltrating tumors. Their immunosuppressive effect is mainly towards T cells, since their high expression of Arginase-I (Arg-I) is believed to deprive T cells in TME of L-arginine which is essential for their function [43,44] (Figure 1). High tissue levels of Arg-I-positive myeloid cells were associated with inferior disease outcomes in HL [45]. Similarly, a subset of MDSCs, circulating CD34^+^ MDSCs, were found to negatively influence the PFS of patients with HL [46]. Interestingly, MDSCs were reduced in patients after treatment with brentuximab vedotin (BV), and baseline serum Arg-I levels emerged as a potential predictive biomarker for BV treatment response [47].

Eosinophils represent one of the most typical cellular populations found in HL biopsies and they are believed to develop a close crosstalk with HRS cells via CD30-CD30L binding, but their prognostic value remains a matter of debate. Although research indicated that eosinophilic tumor infiltration strongly correlates with FFTF, Axdorph et al. did not find any association with clinical outcomes, thus implying the need for further relevant investigation [48,49].

Tumor-associated neutrophils (TANs), similarly to tumor-associated macrophages (TAMs), appear with variable effects in TME, from suppressing anti-tumor immunity to cytotoxicity against neoplastic cells [50]. Due to this and the immunohistochemical overlap with MDSCs, research on the prognostic impact of neutrophils in HL has been limited to their peripheral blood counts rather than tumor infiltration. Indeed, in cHL, a high absolute neutrophil count to a high absolute lymphocyte count ratio is an independent prognostic factor for patients’ reduced OS [51].

### 2.6. Mast Cells

Evidence on the prognostic role of mast cells in HL is controversial, although their biological properties have been well-described. Mast cells are the predominant cells of the TME that express CD30L, the ligand for the CD30 receptor of HRS cells, thus indicating a close interaction with the neoplastic population [52] (Figure 1). Additionally, mast cells are believed to promote tumor growth via the induction of neovascularization and fibrosis, functions that can be inhibited by bortezomib, thus providing a potential therapeutic target [53]. The hypothesized negative prognostic impact, in terms of reduced relapse-free survival, of mast cells in the TME of HL was indeed demonstrated by Molin et al. [54], although other researchers did not find a correlation between mast cell infiltration and prognosis [55].

### 2.7. Dendritic Cells

Dendritic cells (DCs) have also been studied in the TME of HL with variable results, depending on the specific DC subtype. CD123^+^ plasmacytoid DCs are the most abundant DC type in cHL, although they do not seem to correlate with disease-specific survival and they produce reduced amounts of IFN-a compared to healthy individuals, implying an immune functional defect [56,57]. As for myeloid DCs, most of them in cHL TME are identified as a mature CD83^+^ subtype whose number is positively associated with improved disease-specific survival of patients [57]. Finally, the presence of follicular DCs in most subtypes of HL was found to predict a favorable outcome [58]. Patients with cHL were also found to have lower counts of all subtypes of circulating DCs compared to healthy individuals [59].

### 2.8. Tumor-Associated Macrophages

In general, macrophages are derived from mononuclear cells and have multiple roles including, but not limited to, phagocytosis, antigen presentation to other immune cells and tissue remodeling. Among all immune cells found in the TME of cHL, macrophages have attracted the most research interest. This is because of the great plasticity of these cells, indicated by their ability to acquire different phenotypes that influence the tumor microenvironment towards an immunosuppressive or inflammatory state. Accordingly, macrophages variably influence disease progression, and this is probably the reason why the association of TAMs with disease outcomes, treatment response and patients’ survival has been so challenging over the years.

TAMs are recruited in TME through GM-CSF, CCL2, CCL5, CCL7 and CXCL1, which are secreted by neoplastic cells. There, TAMs are programmed towards M1 or M2 phenotypes. Although initially considered as distinct subtypes, this view has been criticized as oversimplified, and the current notion is that M1 and M2 phenotypes actually represent the extremities of a continuum spectrum [60,61].

**Figure 2:**
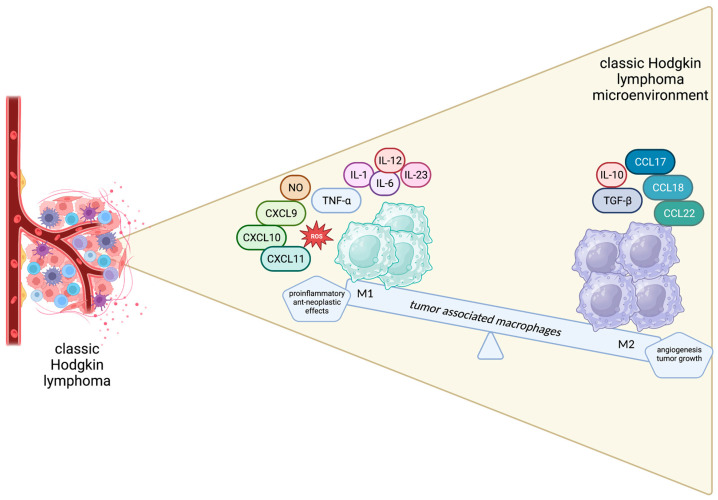
Schematic representation of the spectrum of macrophages in the Hodgkin lymphoma microenvironment.

The M1 phenotype is triggered by GM-CSF, IFN-γ and lipopolysaccharides and is characterized by cytotoxic, pro-inflammatory and anti-neoplastic effects mediated by the secretion of TNF-a, NO, CXCL 9, CXCL10, CXCL11, IL-1, IL-6, IL-12, IL-23 and ROS by M1 macrophages. On the other hand, M2-polarized macrophages, driven by M-CSF, TGF-B, IL-4, IL-10 and IL-13, are believed to promote wound healing, angiogenesis and tumor growth by producing tumor growth factor (TGF)-β, IL-10, CCL17, CCL18, CCL22, CD206, CD204 and CD163 [62]. Apparently, the balance between the anti-neoplastic and pro-tumorigenic phenotypes is crucial for determining disease outcomes and a tempting field for therapeutic interventions (Figure 2).

In cHL, HRS cells can lead TAMs to polarize towards the tumor-promoting M2 phenotype via the secretion of TGF-β and IL-13. In turn, M2 macrophages support the survival of HRS cells, partially through the activation of the STAT3 signaling pathway [62]. As previously discussed, TAMs actively participate in shaping the protective niche around HRS cells and express several immunosuppressive molecules such as IDO-1 and immune checkpoint proteins such as PD-L1 and CD86, through which they interact with immune cells of the TME, further promoting the suppression of anti-tumor immunity [63,64]. Another mechanism in which TAMs might promote tumor growth is supposed to be the induction of the genetic instability of HRS cells, probably through the release of free radicals which contribute to a mutagenic microenvironment. This was based on the observation that TP53 amplification in neoplastic cells, linked with poorer patients’ survival, was associated with increased infiltration by M2 macrophages [65].

Regarding the prognostic implications of macrophage infiltration of cHL, there have been multiple studies with rather contradictory results, probably reflecting the heterogeneity of macrophage sub-populations and the difficulties identifying each macrophage phenotype, given the lack of a definite immunohistochemistry marker. Many available studies usually utilize CD68 as a universal macrophage marker. CD163 is used by other research groups for the further characterization of M2 polarized macrophages, although other researchers doubt whether CD163 is an ideal marker for detecting the M2 subgroup of CD68^+^ macrophages [66].

Steidl et al. were the first to prove that an increased number of TAMs (CD68^+^) is strongly associated with shortened survival in patients with cHL using immunohistochemistry [67]. Similarly, several groups confirmed the negative correlation of CD68^+^ TAMs in HL with patients’ survival and/or response to treatment [68,69,70,71,72,73,74,75], while the prognostic value of CD68^+^ macrophages was not reproduced by other groups [61,76,77,78,79].

When CD163 was used as a TAMs marker, with the rationale of focusing on M2 polarized macrophages, it was also found that increased infiltration correlates with poorer outcomes [73,74,75,78,80] including response to nivolumab [81], but this was doubted by others [79].

In the only meta-analysis conducted concerning the prognostic impact of TAMs in cHL, Guo et al. found that a high density of either CD68^+^ or CD163^+^ TAMs in the TME translates into poorer OS and PFS [82].

Interestingly, Werner et al. demonstrated that both a very high and a very low number of infiltrating TAMs (CD68^+^ or CD163^+^) is associated with worse outcomes, compared with intermediate TAMs levels, while Karihtala et al. indicated that the adverse prognostic effect of TAMs in cHL is immune-checkpoint-dependent, because only PD-L1^+^ and IDO-1^+^ TAMs were associated with inferior outcomes, but not TAMs on the whole [66,83].

Based on the above findings, reprogramming macrophages of the TME in cHL towards the anti-tumorigenic M1 phenotype has been attempted, as in the case of PI3Kδ/γ inhibitor RP6530 [84]. Other therapeutic strategies which exploit TAMs biology with promising results include the inhibition of TAM recruitment in the TME or the direct targeting of TAMs with nanomaterials [60].

### 2.9. CD169^+^ Macrophages: A New Regulator of Antitumor Immunity

CD169^+^ macrophages constitute a subpopulation distinct from M1 and M2 phenotypes, as they can simultaneously express markers of both M1 and M2 subtypes. Normally, they are primarily detected in the metallophilic marginal zone of the spleen and in the medulla and the subcapsular sinus of lymph nodes, but they can also be found in the intestine, liver and bone marrow. Based on their localization, CD169^+^ macrophages (also known as Siglec-1 positive macrophages) basically function as “gatekeepers” of secondary lymphoid organs, since they are the first cell type that captures antigens in lymph nodes and the spleen, present them to other immune cells and, thus, help the activation of T cells and initiate adaptive immune responses. Apart from viral and bacterial inflammatory responses, CD169^+^ macrophages participate in immune tolerance induced by apoptotic cell clearance [85,86]. More interestingly, it has been demonstrated that CD169^+^ macrophages phagocytize dead tumor cells transported via lymphatic flow and present tumor-associated peptides to CD8^+^ T cells, whose cytotoxic activity is augmented, which is considered a crucial step for the induction of antitumor immunity [87,88].

**Figure 3:**
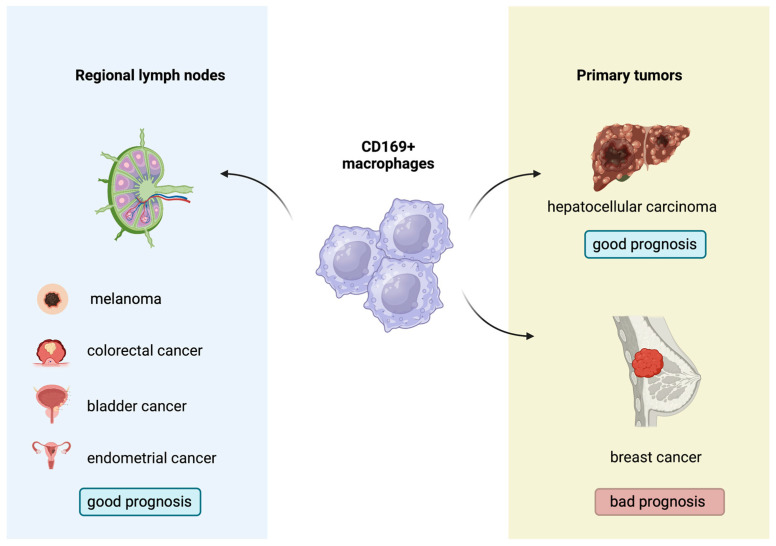
Prognostic role of CD169^+^ macrophages in several solid malignancies.

Indeed, the biological and prognostic role of CD169^+^ macrophages in several human malignancies has gathered research interest. A higher concentration of CD169^+^ macrophages in the primary tumors or regional lymph nodes has been associated with improved outcomes in patients with melanoma, hepatocellular carcinoma, colorectal, bladder and endometrial cancers, with contradictory results in breast cancer [89,90,91,92,93,94,95] (Figure 3). Moreover, Marmey et al. investigated, using immunohistochemistry on paraffin-embedded tissues, the expression of CD169 in 51 cases of B cell non-Hodgkin lymphomas (including diffuse large B cell lymphomas, B-chronic lymphocytic leukemias, follicular lymphomas, mantle cell lymphomas and splenic marginal zone lymphomas). Only splenic marginal zone lymphomas (15 cases) showed a remarkable increase in CD169^+^ cells, with preferential distribution in the splenic cords of the red pulp [96]. These CD169^+^ cells were also positive for CD14 (monocyte/macrophage marker), and it has been hypothesized that the CD169^+^/CD14^+^ cells observed in the splenic cords of splenic marginal zone lymphomas might have a dendritic cell differentiation potential [96]. In this latter study, however, no information about any prognostic implication of the CD169 immunostaining patterns in B cell non-Hodgkin lymphomas was reported [96]. Interestingly, to the best of our knowledge, there are no published studies regarding the potential prognostic impact of the immunohistochemical expression patterns of the CD169^+^ macrophages in the TME of cHL. This investigation could permit a better understanding of the interactions taking place in the TME of cHL and might indicate a new prognostic biomarker or even a therapeutic target, given the already-discussed role of M1 and M2 subpopulations in cHL.

## 3. Immune Evasion

Multiple ways by which HRS cells manage to escape antitumor immune surveillance have been identified. Overall, the neoplastic HRS cells, with their altered gene-expression profiling, seem to hide from antitumor immune cells. On the other hand, as previously discussed, HRS cells attract, through cytokines, several immune cells, which in turn shape an immunosuppressive TME that further allows tumor cells to evade the physiological antitumor immune responses.

At first, HRS cells commonly exhibit copy number gains of 9p24.1 locus, which is associated with the enhanced expression of PD-1 ligands (PD-L1 and PD-L2) in up to 97% of cases. PD-1 ligand expression may also be sustained by EBV infection of HRS cells, independently of 9p24.1 locus alterations [97,98]. These ligands bind to PD-1 receptors on T cells, which inhibits their activation and proliferation, leading to T cell exhaustion. Interestingly, in cHL, PD-L1 is also expressed by TAMs which co-localize with HRS cells, thus augmenting the immunosuppressive signals against host immunity [11,63,99]. HRS cells have been shown to be capable of transferring PD-L1 molecules to TAMs’ surface via trogocytosis, a process that enables neighboring cells to exchange membrane molecules, which underlines the role of HRS cells in actively shaping their microenvironment to their benefit [100]. The immunosuppression via PD-1 interactions has provided the biological rationale for anti-PD1 blockade therapy with nivolumab or pembrolizumab, which, in HL, achieved the highest response rates among human malignancies. Indeed, increased PD-L1 expression on HRS cells has been shown to predict better PFS with PD-1 blockade treatment, functioning as a predictive biomarker [101]. Additionally, the TME of cHL is highly enriched for CTLA-4^+^ T cells, while HRS cells and TAMs provide the corresponding inhibitory ligand, CD86. The interacting T cells and TAMs are gathered around HRS cells, forming a protective niche for the neoplastic cellular population [64]. On the contrary, CD8^+^ cytolytic T cells are located at a distance from this niche, which apparently minimizes their destructive effects on HRS cells [102]. Double therapeutic targeting of the immune checkpoints, CTLA-4 and PD-1, has been proposed as a promising alternative, especially for relapsed or refractory disease. A third important immune checkpoint molecule is LAG-3, expressed on CD4^+^ T cells, NK cells and rarely HRS cells, which acts synergistically with CTLA-4 and PD-1 towards CD8^+^ T cell suppression and the promotion of T_reg_ function. This checkpoint offers an additional target, under investigation, for immunotherapy, alone or in combination with anti-PD-1, since it is considered as one of the major methods of acquired resistance to anti-PD-1 treatment [32,103,104]. A high expression level of all these checkpoint proteins by TME immune cells, mainly CD4^+^ and CD8^+^ T cells and TAMs, is associated with shortened OS, apparently reflecting the immunosuppressive state maintained by these proteins, and this could justify a therapeutic combination of immunotherapy with classic chemotherapy regimens [32]. Another immune checkpoint protein, TIM-3, is generally expressed by innate immune cells and T cells and decreases macrophage activation while promoting MDSCs. In cHL TIM-3 is variably expressed by HRS cells and surrounding immune cells, emerging as an additional target for therapies aiming at restoring the hosts’ anti-tumor immunity by blocking the immunosuppressive checkpoint proteins [105]. As previously discussed, the CXCL13/CXCR5 axis enabling communication between CD4^+^ T cells and B cells in lymphocyte-rich cHL underlines the importance of interplay among non-malignant immune cells in the TME [30] (Figure 1).

HRS cells have also developed means to escape from cytotoxic immune cells. The down-regulation of *B2MG* and MHC-I expression probably allows neoplastic cells to avoid interactions with CD8^+^ T cells. Similarly, inactivating mutations of the *CD58* gene have been detected, especially in cells derived from advanced-stage disease patients. Since CD58 is a receptor recognized by cytolytic CD8^+^ and NK cells, such mutations could further protect HRS cells from immune lysis [106,107].

Extracellular vesicles (EVs) secreted by HRS cells carry TNF-α, ADAM10 and sCD30 that are believed to participate in the education of stromal cells, especially fibroblasts, to support the survival of HRS cells or even protect them from anti-cancer treatments such as BV [108] (Figure 1).

Apart from the direct cellular interactions, the immunosuppressive state of the TME in cHL is largely attributed to the inhibitory function of multiple cytokines and other immune-effective molecules. These include IL-10 and TGF-β produced by Th_2_ CD4^+^ T cells, but also galectin-1, which is mainly produced by HRS cells and exerts multiple inhibitory effects on T cell populations [34]. Histiocytes, dendritic cells and endothelial cells in the TME express IDO [109]. This enzyme catabolizes tryptophan, the depletion of which inhibits T cell function and induces T and NK cell apoptosis, and its expression in the TME of HL, although restricted in specific histologic subtypes, was found to be an independent prognostic factor for inferior survival [34,109,110]. Even the serum kynurenine/tryptophan ratio significantly correlates to OS, further demonstrating the importance of IDO in disease pathophysiology as well as the potential of targeting IDO for a therapeutic benefit [111]. As discussed above, the lymphocytic population of the TME in cHL is highly composed of T_regs_, which have innate immunosuppressive functions as they primarily induce T cell suppression [112].

The interplay between immune cells in TME [30,63,64] is important since it might contribute to the development of immunosuppressive properties of TME. Indeed, the HRS proximal region, also called the neoplastic niche, is usually enriched in PD-1^+^CD4^+^ T cells, which interact with both PD-L1^+^ TAM and PD-L1^+^ tumor HRS cells [63]. In addition, the CTLA4^+^ T_regs_ are also present in the HRS proximal region and interact with TAM exhibiting the PD-L1^+^CD86^+^ immunophenotype [64]. Thus, the immunosuppressive TAMs expressing PD-L1 and possibly CD86 are more frequently found in the close vicinity of tumor HRS cells. The unique topographical distribution of TAM may augment the local source of PD-L1 and likely increases the extent of PD-1 signaling. Moreover, the CXCL13/CXCR5 axis enabling communication between CD4^+^ T cells and B cells in lymphocyte-rich cHL underlines the importance of interplay among non-malignant immune cells in the TME [29].

The cluster of differentiation 47 (CD47) is a molecule that has recently drawn attention as it is also implicated in immune escape mechanisms. CD47, upon binding to signal regulatory protein alpha (SIRPa) that is expressed on macrophages, transduces a “do not eat me” signal (Figure 1). It is presumed that the expression of CD47 in normal cells protects them from phagocytosis [113]. However, its overexpression in cancer cells contributes to their survival advantage since they can evade immune surveillance. CD47 has been found to be overexpressed in 16 cHL cases and especially more intensely in HRS than T cells or stromal cells [114]. In another study with two cohorts, patients with high expression of CD47 on HRS had poorer event-free and overall survival [115] compared to patients with low expression. CD47 has also been found to be an independent, adverse prognostic marker in myeloid malignancies [116] and solid tumors [117]. Clinical trials evaluating the role of magrolimab, TTI-621 or TTI-622 that target and block CD47 in combination with pembrolizumab are ongoing (NCT04788043 and NCT05507541).

## 4. Conclusions

There is a body of evidence showing that the unique TME of cHL is vital for the survival of neoplastic HRS cells as well as tumor growth by providing multiple molecular signals that promote cellular proliferation and protection against a host’s anti-tumor immunity. Each cellular type of the TME, rather than being random bystanders, seems to contribute to forming the immunosuppressive niche for HRS cells, although the whole extent of the molecular interactions among immune cells, or between immune cells and HRS cells, might not be fully elucidated yet.

A possible explanation for the occurrence of aggressive cHL that does not depend on an immunosuppressive environment could be that under selective pressure from the anti-tumor components of the TME, more aggressive HRS tumor cells would eventually be selected, survive and acquire growth advantage [118]. These HRS cells might modify their secretory profile (cytokines, chemokines, etc.), thereby inducing alterations in the cellular composition of the TME. Hence, a more aggressive disease could occur, overcoming, at least in part, the influence of the tumor-suppressive components of the TME. A relevant example of the aforementioned assumption can be recent findings that a subgroup of cHL patients with a high content of HRS cells, suggestive of increased proliferative potential, and a low content of T cells, exhibited an aggressive clinical course [119].

As far as TAMs are concerned, they seem to play a crucial role in shaping the TME and determining the direction of immune responses towards anti-tumor activity or to immunosuppressive and tumor-promoting phenotypes. The exact balance between the two macrophage phenotypes, due to the cellular plasticity of TAMs, is probably the reason for the divergent findings of original studies attempting to correlate TAMs with patients’ survival in cHL, although this might also be attributed to more technical issues, such as defining the most appropriate immunohistochemistry markers.

It is, therefore, reasonable to suggest that more extensive research of macrophage populations, such as the CD169^+^ ones, could shed light on unknown aspects of the TME in cHL and might also provide another prognostic biomarker. Whether this, as well as multiple other previously discussed prognostic biomarkers, will soon be utilized in clinical practice and decision-making remains to be explored, given the complexity of incorporating all these prognostic information into one unified prognostic scoring system.

As far as potential therapeutic implications are concerned, the successful use of immune checkpoint inhibitors has given hope that other sides of tumor immune evasion could be targeted to restore the host’s anti-tumor immunity against lymphoma cells. In this direction, reprogramming the macrophage pool of TME or their monocyte precursors towards the tumor-attacking phenotype, commonly referred to as M1, seems to be a promising strategy. A deeper understanding of the multilevel interactions within the TME of cHL could also guide therapeutic strategies for patients who relapse after standard therapy or are resistant to treatment with immune checkpoint inhibitors. In that way, patients with HL could benefit from more personalized treatments based on their TME to further increase the cure and survival rates of the disease.

## Data Availability

Not applicable.
